# What is a clinical decision analysis study?

**DOI:** 10.4103/0019-5413.40248

**Published:** 2008

**Authors:** Ilyas S. Aleem, Emil H. Schemitsch, Beate P. Hanson

**Affiliations:** Michael G. DeGroote School of Medicine and Division of Orthopaedic Surgery, McMaster University, Hamilton, Ontario, Canada; 1 Division of Orthopaedic Surgery, Toronto, Ontario, Canada; 2 Division of AO-Clinical Investigation, Zurich, Switzerland

**Keywords:** Clinical trials, critical appraisal, decision analysis, evidence-based medicine, hierarchy of evidence

## Abstract

Decision making in clinical practice often involves the need to make complex and intricate decisions with important long-term consequences. Decision analysis is a tool that allows users to apply evidence-based medicine to make informed and objective clinical decisions when faced with complex situations. A Decision Tree, together with literature-derived probabilities and defined outcome values, is used to model a given problem and help determine the best course of action. Sensitivity analysis allows an exploration of important variables on final clinical outcomes. A decision-maker can thereafter establish a preferred method of treatment and explore variables which influence the final outcome. The present paper is intended to give an overview of decision analysis and its application in clinical decision making.

## INTRODUCTION

Making decisions in clinical practice involves a careful analysis of harms and benefits associated with different treatment options. These decisions, often associated with high stakes and important long-term consequences, are frequently made in the face of competing priorities, limited resources and information and an incomplete clinical picture. Under such circumstances, a rigorous and objective analysis of outcomes and probabilities is essential to achieve the best possible decision given a specific clinical situation.

Evidence-based medicine (EBM) refers to the incorporation of critically appraised scientific evidence into clinical practice.^1^,^2^ EBM is arguably the most significant initiative geared towards restructuring clinical practice and reason,^3^ allowing users to integrate both clinical expertise and the best available evidence in the literature.^4^ EBM is now increasingly demanded by clinicians, patients, insurers and even government policy-makers.^5^ Although a randomized clinical trial (RCT) remains the method of choice for establishing the best evidence for a given clinical practice, RCTs may often not be feasible.^5^ This is particularly true in disciplines such as orthopaedic surgery where various surgical procedures and techniques mandate adequate comparison and evaluation.^5^

In the absence of substantiated clinical evidence, many decision-makers have a natural tendency to make overly optimistic, uninformed decisions when faced with complex situations; these choices appear to be made more on the basis of intuition than a rational weighing of outcomes and probabilities.^6^,^7^ Unfortunately, it has been shown that the more complex a decision, the less likely intuition, as opposed to a rigorous analysis of options, will yield positive results.^1^,^6^ This phenomenon, amplified in the clinical setting, has created a need for the application of more objective decision-making techniques, among them being clinical decision analysis.

### Decision analysis and the decision tree

Decision analysis is an objective, explicit method that uses models to represent specific decision problems and allows users to apply EBM to a particular clinical scenario. Factors involved in choosing a given strategy from a group of possible actions are quantitatively evaluated.^1^,^5^ Decision analysis requires the construction of a Decision Tree, which illustrates all plausible relationships, alternatives and outcomes involved with a given decision.^1^,^8^ Associated with each step in the decision tree is a corresponding probability and outcome value. Incorporating both probabilities and outcome values, the decision-analysis model expresses its conclusion in terms of an average expected result.^5^ By using such a tree, a decision-maker can accurately weigh and compare outcomes associated with a given decision, thus leading to a more informed clinical decision.^1^,^9^

Decision analysis is most usefully applied in clinical decisions where there is uncertainty regarding appropriate clinical strategy and when a meaningful tradeoff of advantages and disadvantages is present in the clinical problem.^8^ It must be understood that decision trees are adaptable and that values represent a current and not static, benchmark on which further evolution can be critically evaluated.^1^ Decision analysis models can even be broadened and used by health policy analysts to guide strategies for the care of populations.^5^

### Probabilities and outcome values: What are they and where do they come from?

The basic components to a successful decision analysis are reliable probabilities and outcome values. A probability is a quantitative estimate of the chance or likelihood that a given outcome will occur.^10^ In clinical decision making, probabilities of clinical outcomes can be attained through a systematic and rigorous analysis of available literature, preferably RCTs or other systematic reviews. If there is a deficiency of such literature, researchers must turn to alternatives such as observational studies, expert judgment, existing databases or unpublished work.^10^ These estimated probabilities or baseline probabilities, are then incorporated into the decision tree to assist in the decision making process.

As baseline probabilities may be associated with some degree of uncertainty, reasonable probability ranges must also be specified.^10^,^11^ These ranges can then be used in a sensitivity analysis to assess how different estimates can affect the final decision, as discussed later.^11^

As opposed to probabilities, outcome values are summary measurements of a particular outcome.^10^ They can be expressed in several ways including life years, quality-adjusted life years (QALYs), costs or utilities.^10^ A utility is a measure of a decision maker's relative preference or desirability for a given outcome and is generally expressed as a value between 0 and 1, where 0 is the worst outcome (death) and 1 is the best (perfect health). Utility values can be estimated in several ways, including 1) arbitrary assignment of values based on expert judgment; 2) published values in the literature; or 3) patient preferences.^10^ As with probabilities, the uncertainty of these values can be accounted for by including a range of reasonable values and thereafter performing a scrupulous sensitivity analysis to determine the range of values for which a given outcome is preferred.^11^

### Putting it all together: Calculating the Decision Tree

Once reliable baseline probabilities and outcome values are attained from the literature, expert and/or patient preferences, the tree is ready to be “rolled-back,” or calculated. This is done by multiplying outcome values by their respective probabilities and adding across nodes within a particular decision branch. By rolling back the tree, the model expresses its conclusion in terms of an average expected result, which may be interpreted as life-years, days of treatment, cost or other variables depending on clinical context.^5^ These final values represent baseline values that can then undergo further analysis in the decision tree.

### Sensitivity analysis: Decision Tree hypothesis testing

Although the baseline probabilities and outcome values may show one method to be preferred over another, the difference between options may be quite small. Additionally, baseline probabilities and outcome values are often associated with some uncertainty due to biological variation, differing techniques and expertise and literature discrepancies. As such, a feature of decision analysis, called sensitivity analysis, allows users to perform decision analysis while varying probabilities and outcome values. Sensitivity analysis is the process of repeatedly rolling back the tree with different probability and outcome values, thus allowing users to explore the uncertainty of data and to examine what the effects of variability on probabilities and outcome values in the decision tree have on expected clinical outcomes.^11^ In this process, one or more variables are changed while others are held constant, allowing an exploration of important variables on final outcomes. Sensitivity analysis is a useful method of “debugging,” or identifying errors within a tree; additionally, it is also the decision-makers method of statistical hypothesis testing, allowing the user to assess the degree of uncertainty associated with an analytic result.^11^ Sensitivity analysis thus allows decision trees to be adaptable on which further evolution can be critically evaluated.^1^

### Clinical application of decision analysis

**Figure 1:**
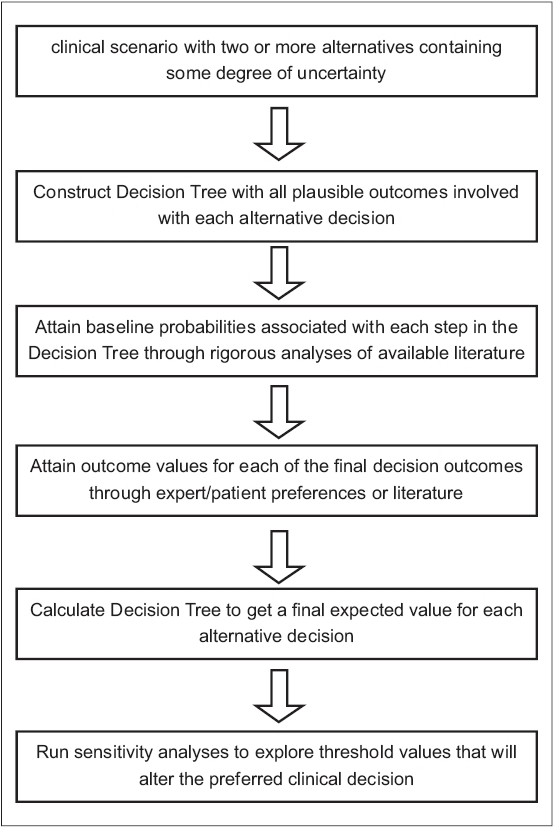
Six steps to a successful clinical decision analysis study

The steps involved in conducting a clinical decision analysis have been summarized in Figure 1. The validity and application of a decision analysis depends entirely on the specific clinical scenario, the availability of data and the strength and inclusion criteria of the selected literature. Furthermore, the results of a decision analysis must be interpreted carefully. Clinicians must look at how closely their particular clinical situation resembles that of the analysis, the strength and reliability of the probabilities and outcome values attained, as well as the results of sensitivity analyses. Such information is then used to result in an informed decision regarding the specific clinical scenario. When it is well executed, incorporating probabilities and outcome values based on accepted data and expert opinion, decision analysis is a powerful tool that has been shown to generate highly credible and reliable results.^1^,^8^,^10^–^12^

Decision analysis has been applied to a number of scenarios of health policy, including management of ventricular septal defects,^1^ screening for prostate cancer^12^ and the treatment of early osteoarthritis of the wrist.^5^

In the management of displaced femoral neck fractures, we recently compared internal fixation and arthroplasty alternatives using a clinical decision analysis. Our model considered the dilemma of the optimal surgical management for an elderly patient with a displaced femoral neck fracture deemed eligible for either prosthetic replacement or internal fixation. We explored the relative benefit of replacement with a prosthesis over internal fixation. Furthermore, we examined the relative outcomes with arthroplasty (total hip versus hemi-arthroplasty) and internal fixation (multiple screws versus sliding hip screws). We aimed to answer which surgical option prevailed when all complications and health states (utilities) were considered. We developed a clinical decision tree with a comprehensive search of the literature and surveys. After analyzing the tree and conducting sensitivity analyses, we found that arthroplasty is favored over internal fixation over a relatively wide range of values, with the most influencing variables being rates of morbidity followed by reoperation.

## SUMMARY

Decision analysis is an objective, explicit method that uses models to represent specific decision problems. A Decision Tree, together with probabilities and outcome values, is used to determine the best course of action. Outcome probabilities are derived from a systematic and rigorous analysis of available literature, preferably RCTs. Outcome values, in the form of life years, QALYs, costs or utilities, are summary measurements of a particular outcome and may be literature derived or from patient/expert opinion. Sensitivity analysis then allows users to explore the effects of variability on important variables and its impact on final clinical outcomes. A decision-maker can thereafter establish a preferred method of treatment and explore variables which influence the final decision. Allowing users to apply EBM to make informed decisions when confronted with difficult scenarios, decision analysis has become a powerful and effective technique with a variety of clinical applications.
